# Southern Dental Association

**Published:** 1886-09

**Authors:** 


### Southern Dental Association.

The annual meeting of this body was held at Nashville, Tennessee, during the last week of July just past, it was one of the best, if not the very best meeting of the body ever held. Quite a large number of members and visitors were in attendance, and the work done was a marked improvement upon that of any other meeting of this Society. A number of excellent papers were read, and very instructive discussions had upon them. One day was devoted to clinics, which was one of the most instructive features of the meeting.

A large number of new and improved appliances, devices, and instruments was exhibited, there were so many good things presented, that it would seem invidious to mention any one in particular, but we feel constrained to mention the appliances, specimens, and practical work of Dr. J. R. Knap, of New Orleans. His specimens of gold crowns, caps and bridgework challenged the admiration of every one who saw them. He exhibited a blow-pipe, which was in reality an oxy-hydrogen blowpipe, very compact, and capable of producing wonderful results, it is quite practicable in all laboratory work. We should be pleased to mention other most excellent appliances did space permit.

The work of the Association under the direction of Dr. J. H. Wardlow was most admirable; would that the Presidents of all our Dental Societies had the tact and the ability of Dr. Wardlow for bringing out of a meeting so large an amount of work.

A full report will appear in the next number of the REGISTER.

